# Explainable and reproducible AI: culturally responsive AI for health equity in minoritized groups

**DOI:** 10.3389/fdgth.2025.1683783

**Published:** 2026-03-25

**Authors:** Michelle King-Okoye, Harriett Fuller, Kuan Tan, Nick Marlow, Jacques Fleuriot, Chris Tzatzakis, Sweekrity Kanodia, Kenneth Odoh, Keerthi Dubbala, Jefferson Alvarez Alvarez

**Affiliations:** 1 Adelaide University, Adelaide, SA, Australia; 2 Health Equity Research Consortium (HERC), London, United Kingdom; 3 Fred Hutch Cancer Center, Seattle, WA, United States; 4 University of Edinburgh, Edinburgh, United Kingdom; 5 Aristotle University of Thessaloniki, Thessaloniki, Greece; 6 Universite Paris Cite, Paris, France

**Keywords:** AI, explainability, reproducibility, ethnic minorities, culturally responsive AI, trust building and communication

## Abstract

Artificial intelligence (AI) is transforming healthcare by enabling advanced diagnostics, personalized treatments, and improved operational efficiencies. By identifying complex data patterns and correlations, AI could supplement clinical decision-making, enabling more rapid diagnoses and treatment decisions tailored to meet the unique needs of diverse communities. However, realizing these benefits requires that clinical AI models be consistent, reliable, and validated across diverse populations and clinical environments. In addition, as these data patterns and correlations may often be unexpected, AI models require more explainability compared to other medical technologies. This is especially true for complex models, where the processes driving a model to make a prediction are often unclear and uninterpretable to both model developers and medical professionals, resulting in AI models frequently being described as “black boxes”. To address this fundamental challenge of interpretability, explainable AI (XAI) has emerged as a critical approach, providing insight (often in a *post-hoc* manner) into why models generate their given output. Studies have shown that most physicians prefer XAI to non-explainable AI. This commentary therefore explores key considerations needed to ensure that AI promotes health equity in marginalized communities, building on similar shifts toward anticipatory health action that have been explored in humanitarian and climate AI contexts (8, 9). We argue that equity in AI depends on embedding explainability and reproducibility within culturally responsive frameworks that address historical and structural bias.

## Introduction

Artificial intelligence (AI) is transforming healthcare by enabling advanced diagnostics, personalized treatments, and improved operational efficiencies (1, 2). By identifying complex data patterns and correlations, AI could supplement clinical decision-making, enabling more rapid diagnoses and treatment decisions tailored to meet the unique needs of diverse communities. However, realizing these benefits requires that clinical AI models be consistent, reliable, and validated across diverse populations and clinical environments. In addition, as these data patterns and correlations may often be unexpected, AI models require more explainability compared to other medical technologies (1, 3). This is especially true for complex models, where the processes driving a model to make a prediction are often unclear and uninterpretable to both model developers and medical professionals, resulting in AI models frequently being described as “black boxes” (1, 4).

To address this fundamental challenge of interpretability, explainable AI (XAI) has emerged as a critical approach, providing insight (often in a *post-hoc* manner) into why models generate their given output (2, 3). Studies have shown that most physicians prefer XAI to non-explainable AI (4, 5). Realistically, complex AI models may not be fully explainable in an accurate manner, resulting in a trade-off between explainability and accuracy (4). Public focus groups have found accuracy to be favored over explainability when AI is implemented in healthcare settings (4). However, further research is needed to ensure that explainability resonates with the realities and values of patients across all communities, particularly minority individuals, whose preferences and needs are often underrepresented in the data used to train AI models (6).

Crucially, when devising explanations in the implementation of XAI, what is considered “explainable” depends on the expertise and expectations of each individual. As such, explanations should be tailored to an individual's expertise and beliefs (5), helping address common concerns that the clinical implementation of AI will amplify existing health disparities in minority populations (2). In addition to XAI, reproducible AI—defined as the ability to replicate results using the same data, code, and methodologies—plays a pivotal role in ensuring that AI advances benefit all (7).

Reproducibility is not merely a technical benchmark but also a safeguard for equity, ensuring that AI performs reliably across clinical contexts and diverse population groups. Taken together, explainability and reproducibility are necessary but not sufficient. They must be applied through a culturally responsive lens, recognizing that trust, acceptance, and equity are shaped by social context, lived experience, and historical patterns of exclusion. This commentary therefore explores key considerations needed to ensure that AI promotes health equity in marginalized communities, building on similar shifts toward anticipatory health action that have been explored in humanitarian and climate AI contexts (8, 9). We argue that equity in AI depends on embedding explainability and reproducibility within culturally responsive frameworks that address historical and structural bias.

## Patient perception and trust in minority communities

Individuals from minority communities often experience heightened mistrust in the medical profession due to historical atrocities and systemic racism. As demonstrated during the COVID-19 vaccination campaigns (6, 10), a failure to adequately address these specific sources of mistrust minimizes health service utilization by minority populations, exacerbating existing health disparities (6, 8). Challenges arise due to limited data representation of marginalized and minority communities and the inability to validate the results using independent datasets. To address this mistrust, XAI's explanatory capabilities should be tailored to individual population groups, addressing key sources of mistrust of each group while accounting for beliefs, language barriers, medical literacy, resource access, and the cultural importance of family members in medical decision-making. This will increase community participation, which will be beneficial to data representativeness.

In addition to mistrust, reduced patient autonomy is a key concern in the ethical implementation of AI in clinical settings, particularly among minority populations (9). If medical professionals are unable to clearly explain AI-informed clinical decisions, or if patients are unable to understand these explanations, patients are likely to have less autonomy when making healthcare decisions. This reduced autonomy compromises their ability to provide informed consent (1). Moreover, opportunities to partake in shared decision-making (SDM) and preferences around SDM are not always consistent across minority communities and should be explored further in relation to XAI across diverse populations (7). Another key concern in the implementation of AI is data privacy. Although applicable to all patients, data privacy concerns may be heightened among marginalized patients with lower trust in medical and government systems. These patients may experience greater levels of fear regarding the accidental de-identification of sensitive data by algorithms (9). As clinical AI use expands, it is imperative that AI implementation studies are designed in a culturally sensitive manner and are conducted across diverse population groups, incorporating all perspectives on mistrust, autonomy, and privacy. Indeed, insights from recent and ongoing work on culturally grounded silence and stigma in prostate cancer awareness among Afro-Caribbean men and First Nations communities highlight how cultural beliefs can be implemented to guide AI health innovations (11).

This example illustrates how cultural responsiveness and explainability intersect: AI innovations must not only provide clear reasoning but also demonstrate reliability across groups while respecting community-specific beliefs about illness, family, and confidentiality. In some communities, discussions of illness are often confined to trusted family networks, with public acknowledgment discouraged by cultural expectations of self-censure and concealment of vulnerability. When applied to AI health innovations, these dynamics suggest that explainability cannot rely only on technical reasoning but must also be framed in ways that respect community communication patterns and emphasize confidentiality (11). AI systems introduced with culturally attuned explanations—such as those that acknowledge the role of family in decision-making and address concerns about stigma—are more likely to build trust and encourage engagement. These same issues of trust and understanding also shape how healthcare professionals interpret AI systems, influencing the success of XAI in practice.

## Acceptance and understanding by healthcare professionals

Similar to patients, medical professionals have also expressed mistrust in AI implementation (12). Fostering acceptance of AI in healthcare requires a nuanced approach that identifies the sources of mistrust, acknowledges the beliefs and perceptions of medical professionals, accommodates varying levels of AI readiness and knowledge, and positions community voices at the forefront of discussions.

Mistrust in AI implementation among healthcare professionals has been shown to strongly correlate with the explainability of AI models (10, 12). Although fully explainable complex AI models may not be a reality, models that medical professionals can independently verify could improve trust (5).

Continual, tailored training and explanation of models are more feasible solutions to improving explainability and the acceptance of AI by healthcare professionals, while also addressing concerns surrounding reduced autonomy, professional identity, and liability (10, 12, 13). In addition to the development, implementation, and strengths and limitations of AI, this training should focus on bias mitigation strategies and health system integration, allowing providers to appropriately understand model outputs and communicate them clearly to patients (1, 3, 14).

An emphasis on the development of clinical reasoning and critical thinking skills will also be essential to ensure successful XAI implementation. These skills address concerns about maintaining professional identity, autonomy, and expertise, the diminishment of which have been positioned as key sources of mistrust in AI among healthcare professionals. Clinical reasoning and critical thinking skills have been identified as the most important factors in determining the accuracy of clinical decisions, but they remain key components of decision-making that are lacking in generative AI models.

Generative AI models have demonstrated the ability to provide clinical recommendations that align more closely with expert guidelines than those provided by emergency and triage nurses. However, these recommendations are more conservative and less varied, highlighting the importance of integrating of human clinical reasoning skills into clinical decision-making. Importantly, concerns have been raised regarding the diminishment of critical thinking skills and clinical judgment following widespread AI implementation. Care must be taken to ensure that the implementation of AI itself does not negatively impact the training and learning of healthcare staff (14, 15).

Finally, for XAI to be accepted and trusted by healthcare professionals, it is essential that concerns surrounding accountability and liability are addressed (5). Even if individuals believe they can autonomously explain, understand, and implement AI diagnostic decisions, they are still responsible for any healthcare decision made. It is therefore imperative to further investigate the perspectives of medical professionals from minority backgrounds regarding liability, who may have already experienced workplace discrimination (11), particularly if AI implementation affects beliefs about autonomy and mistrust among both patients and providers. Yet ensuring clinicians trust AI is not enough unless models themselves remain reproducible and equitable. Without parallel investment in professional autonomy training, explainability alone cannot prevent perceived deskilling, which may ultimately hinder adoption.

## Importance of reproducible AI for AI equity

Reproducible AI is critical for ensuring consistency and validation in healthcare, where AI models must perform reliably across diverse clinical settings and population groups. By standardizing processes, enabling robust testing, and facilitating version control, reproducible AI ensures consistency in model development. This approach enables verification of results and independent model validation across populations while also holding developers accountable for reducing the risk of errors or biases (14, 15). For healthcare institutions, reproducibility provides the evidence needed to adopt AI solutions confidently, ensuring patient safety and regulatory compliance (16). Although in practice, challenges surrounding data privacy, transparency, and resource constraints must be overcome to fully realize the benefits of reproducible AI in healthcare, reproducible AI will likely help improve trust in AI and the performance of AI across diverse population groups.

Furthermore, healthcare datasets are inherently diverse, reflecting variations in patient demographics, clinical practices, and data collection methods. This means it is essential that minority populations are adequately represented at all stages of model development and assessment to ensure that models perform effectively across population groups. Community-based participatory research offers significant potential to embed diverse lived experiences into the design and development of AI systems. Involving the community as partners ensures that the technology reflects their values, addresses their priorities, and fosters trust and sustained engagement (17). Here, explainability and reproducibility intersect: Explanatory tools can reveal when models depend on fragile or biased features, while reproducibility testing across diverse datasets ensures these weaknesses are corrected. Cultural responsiveness then ensures that validation processes are sensitive to social context, not just statistical performance.

It is well established that minority populations remain underrepresented in biological datasets, including genetic data, multi-omics data, and medical imaging repositories (2, 18). To prevent the worsening of health disparities and ensure that AI is effective (and trusted) in all populations, efforts must continue to recruit and include diverse participants in medical research studies, repositories, and biobanks. Following increased data collection and generation, the adoption of reproducible AI approaches will facilitate robustness testing across multiple datasets and institutions. This further ensures that models generalize effectively to different populations and settings (19). Technique-wise, hybrid approaches such as Neurosymbolic AI (20)—which enables gathered knowledge to be embedded, for example, as rules during model construction—should be explored to mitigate data scarcity. Furthermore, reproducible AI will also aid in transparent multi-site validation evaluation, enabling independent researchers to verify model performance and identify potential biases (7). Multi-site validation, as emphasized by regulatory frameworks like the FDA's Software as a Medical Device (SaMD) guideline, ensures that models perform reliably across diverse clinical environments (13). When coupled with culturally responsive validation, reproducibility thus functions as both a technical requirement and an equity safeguard, ensuring that AI performs reliably for all.

## AI implementation: a global perspective

AI has shown promise in supporting healthcare for both communicable and non-communicable diseases in low- and middle-income countries. However, challenges remain in integrating these successes into clinical settings, despite fieldwork conducted before and after model development (17). Unique challenges in resource-limited settings—including connectivity issues, limited infrastructure, and poor access to medical equipment (e.g., digital and imaging technologies)—should be carefully addressed to ensure successful global AI implementation. Furthermore, the knowledge of medical providers and community health workers should also be considered to ensure that model limitations are not overlooked when implementing AI approaches in clinical settings. To achieve this, community involvement must be a core focus at all stages of model development and implementation, with models trained and validated in the target population group. AI should also address medical research priorities relevant to low- and middle-income countries, while accounting for regional differences in data privacy regulations (2, 9). In high-income countries, discrepancies in healthcare access to providers with required technologies and expertise must also be considered in AI implementation and validation. For example, AI models that use cost as a proxy for healthcare need have been shown to inadequately consider the needs of Black individuals due to fewer interactions with the healthcare system, resulting in lower healthcare costs for this population (21).

## Conclusion

Collectively, the use of XAI and reproducible AI will be essential for improving trust in AI and successful AI implementation across population groups, ensuring that models perform reliably across diverse settings. While challenges remain, addressing these barriers is critical for realizing the full potential of AI in healthcare while minimizing risks and ensuring ethical, equitable, and reliable care. The incorporation of a wide range of perspectives and expertise in the development and initiation of AI programs, combined with continuous evaluation and validation across diverse clinical settings, will help ensure that the AI revolution in healthcare benefits all communities equitably. Moreover, ensuring community voices are central to AI systems—from design to implementation—will be key to creating positive impact (17).

## Data Availability

The original contributions presented in the study are included in the article/Supplementary Material; further inquiries can be directed to the corresponding author.

## References

[1] Chan B. Black-box assisted medical decisions: AI power vs. ethical physician care. Med Health Care Philos. (2023) 26(3):285–92. 10.1007/s11019-023-10153-z 37273041 PMC10425517

[2] Marko JGO Neagu CD Anand PB. Examining inclusivity: the use of AI and diverse populations in health and social care: a systematic review. BMC Med Inform Decis Mak. (2025) 25(1):57. 10.1186/s12911-025-02884-1 39910518 PMC11796235

[3] Theunissen M Browning J. Putting explainable AI in context: institutional explanations for medical AI. Ethics Inf Technol. (2022) 24(2):23. 10.1007/s10676-022-09649-8 35539962 PMC9073821

[4] van der Veer SN Riste L Cheraghi-Sohi S Phipps D Tully M Bozentko K Trading off accuracy and explainability in AI decision-making: findings from 2 citizens’ juries. J Am Med Inform Assoc*.* (2021) 28(10):2128–38. 10.1093/jamia/ocab127 34333646 PMC8522832

[5] Abgrall G Holder AL Chelly Dagdia Z Zeitouni K Monnet X. Should AI models be explainable to clinicians? Crit Care. (2024) 28(1):301. 10.1186/s13054-024-05005-y 39267172 PMC11391805

[6] Obermeyer Z Powers B Vogeli C Mullainathan S. Dissecting racial bias in an algorithm used to manage the health of populations. Science. (2019) 366(6464):447–53. 10.1126/science.aax2342 31649194

[7] Haibe-Kains B Adam GA Hosny A Khodakarami F Thakkar S Kusko R Transparency and reproducibility in artificial intelligence. Nature. (2020) 586(7829):E14–6. 10.1038/s41586-020-2766-y 33057217 PMC8144864

[8] Fuller H Dubbala K Obiri D Mallare M Advani S De Souza S Addressing vaccine hesitancy to reduce racial and ethnic disparities in COVID-19 vaccination uptake across the UK and US. Front Public Health. (2021) 9:789753. 10.3389/fpubh.2021.789753 34950633 PMC8688686

[9] King-Okoye M. (2025). Breaking the silence around prostate cancer for black African and Indigenous communities. Aust Nurs Midwifery J. 28(7):34–5. Available online at: https://search.informit.org/doi/10.3316/informit.T2025061800007701111276769

[10] Levin C Zaboli A Turcato G Saban M. Nursing judgment in the age of generative artificial intelligence: a cross-national study on clinical decision-making performance among emergency nurses. Int J Nurs Stud. (2025) 172:105216. 10.1016/j.ijnurstu.2025.105216 40975905

[11] Perez Jolles M Richmond J Thomas KC. Minority patient preferences, barriers, and facilitators for shared decision-making with health care providers in the USA: a systematic review. Patient Educ Couns. (2019) 102(7):1251–62. 10.1016/j.pec.2019.02.003 30777613

[12] Ackerhans S Wehkamp K Petzina R Dumitrescu D Schultz C. Perceived trust and professional identity threat in AI-based clinical decision support systems: scenario-based experimental study on AI process design features. JMIR Form Res. (2025) 9:e64266. 10.2196/64266 40138691 PMC11982750

[13] Jussupow E Spohrer K Heinzl A. Identity threats as a reason for resistance to artificial intelligence: survey study with medical students and professionals. JMIR Form Res. (2022) 6(3):e28750. 10.2196/28750 35319465 PMC8987955

[14] Panahi S Spearman B Sundrud J Lunceford M Kamimura A. The impact of patient autonomy among uninsured free clinic patients. J Patient Exp. (2023) 10:23743735231179041. 10.1177/23743735231179041 37323759 PMC10265317

[15] Okolo CT. Optimizing human-centered AI for healthcare in the global south. Patterns. (2022) 3(2):100421. 10.1016/j.patter.2021.100421 35199066 PMC8848006

[16] Diprose WK Buist N Hua N Thurier Q Shand G Robinson R. Physician understanding, explainability, and trust in a hypothetical machine learning risk calculator. J Am Med Informatics Assoc. (2020) 27(4):592–600. 10.1093/jamia/ocz229 PMC7647292 32106285

[17] Kelly CJ Karthikesalingam A Suleyman M Corrado G King D. Key challenges for delivering clinical impact with artificial intelligence. BMC Med. (2019) 17(1):195. 10.1186/s12916-019-1426-2 31665002 PMC6821018

[18] Topol EJ. High-performance medicine: the convergence of human and artificial intelligence. Nat Med. (2019) 25(1):44–56. 10.1038/s41591-018-0300-7 30617339

[19] Kendrick A Krishnan N Baharani J Tuttle J Szczepura A. Gender, race and ethnicity biases experienced by hospital physicians: an umbrella review to explore emerging biases in the evidence base. BMJ Open. (2025) 15(2):e094549. 10.1136/bmjopen-2024-094549 39956599 PMC11831289

[20] DeLong LN Mir RF Fleuriot JD. Neurosymbolic AI for reasoning over knowledge graphs: a survey. IEEE Trans Neural Networks Learn Syst. (2025) 36(5):7822–42. 10.1109/TNNLS.2024.3420218 39024082

[21] Jiang F Jiang Y Zhi H Dong Y Li H Ma S Artificial intelligence in healthcare: past, present and future. Stroke Vasc Neurol. (2017) 2(4):230–43. 10.1136/svn-2017-000101 29507784 PMC5829945

